# Exosomal miR-106b-5p derived from melanoma cell promotes primary melanocytes epithelial-mesenchymal transition through targeting EphA4

**DOI:** 10.1186/s13046-021-01906-w

**Published:** 2021-03-19

**Authors:** Wenkang Luan, Yuting Ding, Haolan Xi, Hongru Ruan, Feng Lu, Shaojun Ma, Jinlong Wang

**Affiliations:** 1 Department of Plastic Surgery, Affiliated People’s Hospital of Jiangsu University, 8 Dianli Road, Zhenjiang, 212000 Jiangsu China; 2 Department of Rehabilitation, Changshu No. 2 People’s Hospital (The 5th Clinical Medical College of Yangzhou University), Changshu, Jiangsu China; 3 Department of Ophthalmology, Affiliated People’s Hospital of Jiangsu University, Zhenjiang, Jiangsu China

**Keywords:** Melanoma, Exosomal, miR-106b-5p, EphA4, EMT

## Abstract

**Background:**

Cancer-secreted exosomal miRNAs regulates the biological processes of many tumours. The serum level of exosomal miR-106b-5p is significantly increased in melanoma patients. However, the role and molecular mechanisms of exosomal miR-106b-5p in melanoma remains unclear.

**Methods:**

Quantitative real-time polymerase chain reaction (qRT-PCR) was used to detect the expression of miR-106b-5p and EphA4 in melanoma tissues. Transmission electron microscopy (TEM) and western blotting were used to identify exosome. QRT-qPCR and Cy3-labelled miR-106b-5p were used to demonstrated the transmission of melanoma cell-secreted exosomal miR-106b-5p. Western blotting, Immunofluorescence, adhesion, transwell and scratch wound assay were used to explore the role of exosomal miR-106b-5p in melanocytes. Luciferase reporter assays and RNA-Chromatin Immunoprecipitation (ChIP) assay were used to confirm whether erythropoietin-producing hepatocellular carcinoma receptor A4 (EphA4) was a direct target of miR-106b-5p.

**Results:**

We found that miR-106b-5p levels were increased in melanoma tissue, and high miR-106b-5p expression is an independent risk factor for the overall survival of patients with melanoma. miR-106b-5p is enriched in melanoma cell-secreted exosomes and transferred to melanocytes. Exosomal miR-106b-5p promotes the epithelial-to-mesenchymal transition (EMT), migration, invasion and adhesion of melanocytes. Exosomal miR-106b-5p exerted its role by targeting EphA4 to activate the ERK pathway. We demonstrated that exosomal miR-106b-5p promoted melanoma metastasis in vivo through pulmonary metastasis assay.

**Conclusions:**

Thus, melanoma cell-secreted exosomal miR-106b-5p may serve as a diagnostic indicator and potential therapeutic target in melanoma patients.

**Supplementary Information:**

The online version contains supplementary material available at 10.1186/s13046-021-01906-w.

## Background

Malignant melanoma is the most aggressive skin cancer and its global incidence is increasing every year [1–3]. It has been reported to be the main cause of skin tumour-related death [4]. Although surgery is the first treatment for the primary stage of melanoma, the survival of patients with metastatic dissemination is significantly reduced [5]. Therefore, it is important to understand the molecular mechanisms underlying melanoma progression and metastasis.

Exosomes, which are 30–150 nm endocytic vesicles, are produced and released by a variety of cells, including tumour cells [6, 7]. Exosomes have been implicated in intercellular communication by transmitting different molecules, including proteins, lipids, and functional RNA molecules, from donor cells to recipient cells [8, 9]. miRNAs, which are 20–22 nucleotide non-coding RNA, regulate gene expression by cleaving or inhibiting the translation of target mRNAs [10, 11]. Cancer-secreted exosomal miRNAs can be transferred into recipient cells to regulate target genes and biological processes of tumours [12, 13]. Melanoma cells release a large number of exosomes, and substantial differences in the profiles of exosomal miRNAs in the plasma of patients with melanoma have been observed [14, 15]. Nevertheless, only a few exosomal miRNA functions have been identified in melanoma [12, 16]. It has been reported that exosomal miR-106b-5p in serum is present at significantly higher levels in patients with melanoma [16]. However, the function and molecular mechanisms of exosomal miR-106b-5p during melanoma development remains unknown.

Melanoma is a cancer that originates from melanocytes. Although melanocytes do not belong to the epithelial cell line, primary melanocytes express E-cadherin [17]. Normal melanocytes also express epithelial-to-mesenchymal transition (EMT)-inducing transcription factors (EMT-TFs), which are intrinsic factors that lead to the high metastatic propensity of melanoma [17]. The EMT process contributes to the progression and metastasis of melanoma [18], but there are few studies on the effect of melanoma exosomes on the progression of melanoma by affecting EMT in tumour microenvironment. EphA4, which is a member of the large Eph (erythropoietin-producing hepatocellular carcinoma) family of receptor tyrosine kinases, has been shown to play oncogenic and tumour-suppressive roles in many human tumours [19, 20]. EphA4 effectively suppresses the EMT and metastatic capabilities of melanoma cells by interfering with the activation of ERK [21].

In this study, we showed that miR-106b-5p expression is increased in melanoma tissue and that high miR-106b-5p expression is an independent risk factor for patients with melanoma. Moreover, miR-106b-5p was also shown to be highly enriched in exosomes secreted from melanoma cells. Melanoma cell-derived exosomal miR-106b-5p promotes melanocyte EMT by directly repressing EphA4 to activate ERK pathways, leading to the establishment of a tumour metastasis-supporting microenvironment. Thus, exosomal miR-106b-5p derived from melanoma cells may serve as a diagnostic indicator and potential therapeutic target in patients with melanoma.

## Materials and methods

### Human tissues

We collected 36 primary malignant melanoma tissues and adjacent normal tissues from the Affiliated People’s Hospital of Jiangsu University. Samples were stored in liquid nitrogen after collection, and the clinicopathological features of the tissue were independently diagnosed by two pathologists. All patients received no chemotherapy or radiotherapy before operation. Serum samples of peripheral blood from patients with primary and 12 metastatic melanoma were also collected in the Affiliated People’s Hospital of Jiangsu University. This study protocol was approved by the Human Research Ethics Committee of the Affiliated People’s Hospital of Jiangsu University. The informed consent was obtained from each participant. Melanoma cases from The Cancer Genome Atlas (TCGA) and the Gene Expression Omnibus (GEO#GSE34460 and GEO#GSE24996) were also included in this study.

### Cell culture

The A375 human malignant melanoma cell line was obtained from the Chinese Academy of Sciences Cell Bank (Shanghai, China), and the A2058, SK-MEL-1 and SK-MEL-28 human malignant melanoma cell lines were obtained from the American Type Culture Collection. The cells were cultured in Dulbecco’s modified Eagle’s medium (DMEM; Gibco, USA), which contained 10% foetal bovine serum (FBS; Invitrogen, USA). HEMa-LP human epidermal melanocytes were purchased from Invitrogen and grown in medium 254 (Cascade Biologics).

### Oligonucleotides, plasmids and transfection

miR-106b-5p mimic, miR-106b-5p inhibitor and negative controls were chemically synthesized by GenePharma (Shanghai, China), and the small interference RNAs (siRNAs) for the reduceion of EphA4 expression were also obtained from GenePharma. EphA4 was amplified in full length and inserted into pcDNA3.1 vectors (Invitrogen, USA) to construct EphA4 plasmid. The lentiviral empty vector and miR-106b-5p inhibitor vector were also obtained from GeneChem (Shanghai, China). Melanoma cells were infected with lentiviruses in order to obtain stably expressing miR-106b-5p inhibitor cells. The oligonucleotides and plasmids were transfected into melanoma cells or melanocytes via Lipofectamine 2000 (Invitrogen, USA) according to the manufacturer’s instructions.

### Extraction of RNA and quantitative RT-PCR

Total RNA was extracted from cells and tissues by using TRIzol reagent (Invitrogen, USA). Reverse transcription (RT) was performed using Fermentas reverse transcription reagents and an Applied Biosystems® TaqMan® MicroRNA Reverse Transcription Kit (Applied Biosystems, CA). An ABI StepOnePlus system (Applied Biosystems, CA) was used for amplification reactions according to the predetermined conditions. To analyse the expression of miR-106b-5p, the primer for miR-106b-5p was purchased from RiboBio (Guangzhou, China), and U6 was used for normalization. The primer for miR-106b-5p were 5′-TGCGGCAACACCAGTCGA TGG-3′ and 5′-CCAGTGCAGGGTCCGAGGT-3′. The primer for U6 were 5′-CTCGCTTCGGGCAGCACA-3′ and 5′-AACGCTTCACGAATTTGCGT-3′. To analyse the expression levels of EphA4, GAPDH was used for normalization. The primers for EphA4 were 5′-CAGAGGTAAGGGTAGGAGGC-3′ and 5′-AGCAGTGTAGCGAGCACAAC-3′. GAPDH forward, 5′-AACTTTGGCATTGTGGAAGG-3′ and reverse, 5′-GGATGCAGGGATGATGTTCT-3′. The data were calculated using the 2^–△△Ct^ method.

### Western blot analysis

Total protein from cells, tissues and exosomes was extracted with RIPA buffer (Kengen, China) and quantitatively analysed with a BCA Protein Assay Kit (Beyotime, China). Western blotting was conducted as previously described [22]. Antibodies against CD63, TSG101, calnexin, fibronectin, EphA4, Snail, total ERK, and p-ERK^T202/Y204^ were purchased from Abcam (Cambridgeshire, UK). Antibodies against E-cadherin, N-cadherin, ß-actin and GAPDH were purchased from Cell Signalling Technology (CST, USA).

### Luciferase reporter assay

The 3′-UTR fragment of EphA4 containing the binding site of miR-106b-5p was cloned into the pMIR-REPORT vector, and mutant plasmids were used as a control. The miR-106b-5p mimic and related reporter plasmids were co-transfected into melanocytes. A Dual Luciferase Reporter Assay Kit (Promega, USA) was used to analyse the luciferase activity after 48 h according to the manufacturer’s instructions.

### Isolation of RISC-associated RNA

HEMa-LP cells overexpressing miR-106b-5p or NC were fixed with 1% formaldehyde, and chromatin fragments were then processed. The cells were lysed in NETN buffer and incubated with Dynabeads Protein A (Invitrogen, USA) supplemented with IgG or the anti-Pan-Ago clone 2A8 antibody (Millipore, USA). Immunoprecipitated RNA was released by proteinase K digestion. Phenol/chloroform/isopropyl alcohol was used to extract RNA. RNA was purified by glycogen ethanol precipitation, resolved and treated with DNase I.

### Exosome isolation, labelling and identification

Malignant melanoma cells were cultured in DMEM supplemented with 10% exosome-depleted FBS for 48 h. Ten millilitres of cell conditioned medium or 250 μl serum (peripheral blood from patients with primary and metastatic melanoma) was mixed with ExoQuick exosome precipitation solution (System Biosciences, USA). Exosome isolation was conducted using ExoQuick-TC™ (System Biosciences, USA) according to the manufacturer’s protocol. For transmission electron microscopy, the exosomes were loaded onto carbon-coated 300 mesh copper grids (Agar Scientific Ltd., Stansted, UK), fixed with 3% glutaraldehyde and 1% osmium tetroxide, and then stained with uranyl acetate and lead citrate. The exosomes were observed by Transmission electron microscopy (TEM, Hitachi, Japan) after air-drying. For RNA extraction from the exosomes, a miRNeasy Mini Kit (Qiagen) was used. The concentration of exosomes protein was determined by using a BCA Protein Assay Kit (Beyotime, China). A PKH67 Green Fluorescent Cell Linker Mini Kit (Sigma, USA) was used to label the purified exosomes according to the manufacturer’s instructions. Images were acquired with a confocal microscope.

### Immunofluorescence (IF) assay

HEMa-LP cells were seeded on collagen-coated glass coverslips and fixed in 4% formaldehyde after washing with phosphate-buffered saline (PBS) at the time of cell harvest. The cells were permeabilized with 0.1% Triton X-100 and blocked with 1% BSA in PBS to prevent non-specific binding. The cells were treated with antibodies against N-cadherin (Abcam, Cambridgeshire, UK), then incubated with a secondary Fluor594 -conjugated goat anti-mouse IgG (Jackson, 1:100 dilution) antibody, and post-stained with DAPI. Fluorescence microscopy was used to photograph the fluorescence images.

### Detection of Cy3-labelled miR-106b-5p transfer

Melanoma cells were transfected with Cy3-labelled miR-106b-5p (GenePharma, Shanghai, China) or miR-106b-5p without Cy3-labelling. Then, HEMa-LP cells were cultured with exosomes (10 μg of exosomes resuspended in 100 μl PBS) purified from the above mentioned treatment groups. HEMa-LP cells were stained with PKH67 and DAPI, and images were acquired with a confocal microscope.

### Cell invasion, migration and adhesion assays

For the transwell assay, HEMa-LP cells were resuspended in serum-free DMEM and placed on top of Matrigel-coated chambers (BD Biosciences, USA). DMEM containing 10% foetal bovine serum was added to the lower chamber as a chemoattractant. After 24 h, we used cotton swabs to remove non-invasive cells. The invasive cells were fixed, stained with 0.1% crystal violet, counted and imaged using a microscope (100× magnification). For the scratch wound assay, HEMa-LP cells were seeded into 6-well plates, and a 200 μl pipette tip was used to form wound gaps after transfection for 24 h. At 0 and 48 h, the cells were imaged under a microscope to analyse the wound width. ImageJ software was used for imaging analysis. For the adhesion assay, the 96-well plates were pre-coated with fibronectin (100 μg/ml, Sigma, USA) overnight at 4 °C. Plates were blocked with 1% BSA for an additional 2 h at 37 °C. A total of 2 × 10^4^ HEMa-LP cells were added to each well and incubated for 40 min. Non-adhesive cells were then removed using PBS. The adhesive cells were fixed and stained with 0.1% crystal violet, and counted and photographed using a microscope.

### Fluorescence in Situ Hybridization (FISH)

The expression of miR-106b-5p in 5 human malignant melanoma samples and adjacent normal tissues was analysed by FISH. FISH was carried out by using a RiboTM Fluorescent In Situ Hybridization Kit (RiboBio, China) as previously described [23]. Nuclear signals and all signals in the positive sites of the miR-106b-5p probes were counted in different fields of view of the tissue, and 3 fields of view were selected for each group. The FISH results were calculated as follows: FISH result = probe positive signal / nuclear signal. ImageJ software was used to collect the signals.

### In vivo pulmonary tumour metastasis assay

Mice were purchased from the Beijing Laboratory Animal Center (Beijing, China). A375 (1 × 10^6^ cells in plain DMEM) cells stably transfected with the control lentiviral vector or miR-106b-5p inhibitor vector were injected into the tail vein of the mice. The mice were randomly assigned into groups and injected intravenously with 20 μg of exosomes secreted by miR-NC- or anti-miR-106b-5p-transfected A375 cells 3 times at 48 h intervals. miR-106b-5p inhibitor lentiviral vector-transfected A375 cells were injected via the tail vein after the exosome treatment. The mice were anaesthetized before an intraperitoneal injection of sterile D-luciferin Firefly potassium salt solution (30 mg/mL) after 4 weeks. Tumour cells colonized in the lung were identified by bioluminescent signals, which were obtained using a Xenogen IVIS 200 instrument (Xenogen, CA) for 4 min of in vivo imaging. The results were quantified as the average radiance of photons emitted per second and area by using Living Image software (Xenogen, CA). The mice were sacrificed. Then, the lungs were dissected, and the metastatic nodules were counted. This study was approved by the Experimental Animal Ethics Committee of the Affiliated People’s Hospital of Jiangsu University.

### Immunohistochemistry staining and hematoxylin-eosin (HE) staining

Immunohistochemistry staining was performed as described previously [24] using antibody against EphA4 (Abcam, Cambridgeshire, UK). For HE staining, sections were incubated with hematoxylin solution after deparaffinization and rehydration. The sections were stained with five dips in 1% acid ethanol and eosin solution. Graded alcohol was used to dehydrate the sections followed by clearing in xylene. The representative images were taken by fluorescence microscope.

### Statistical analysis

The data are expressed as the mean ± standard deviation (S.D.), and SPSS 13.0 (IBM, NY, USA) was used to analyze the data. A t-test or one-way ANOVA was used to evaluate statistical significance. Spearman correlation analysis was carried out using MATLAB, and survival plots were drawn using Kaplan-Meier analysis. *P* < 0.05 was considered to have statistical significance, and *P* < 0.01 had strongly significant.

## Results

miR-106b-5p expression is upregulated in melanoma and an independent risk factor for the survival of patients with melanoma.

**Fig. 1:**
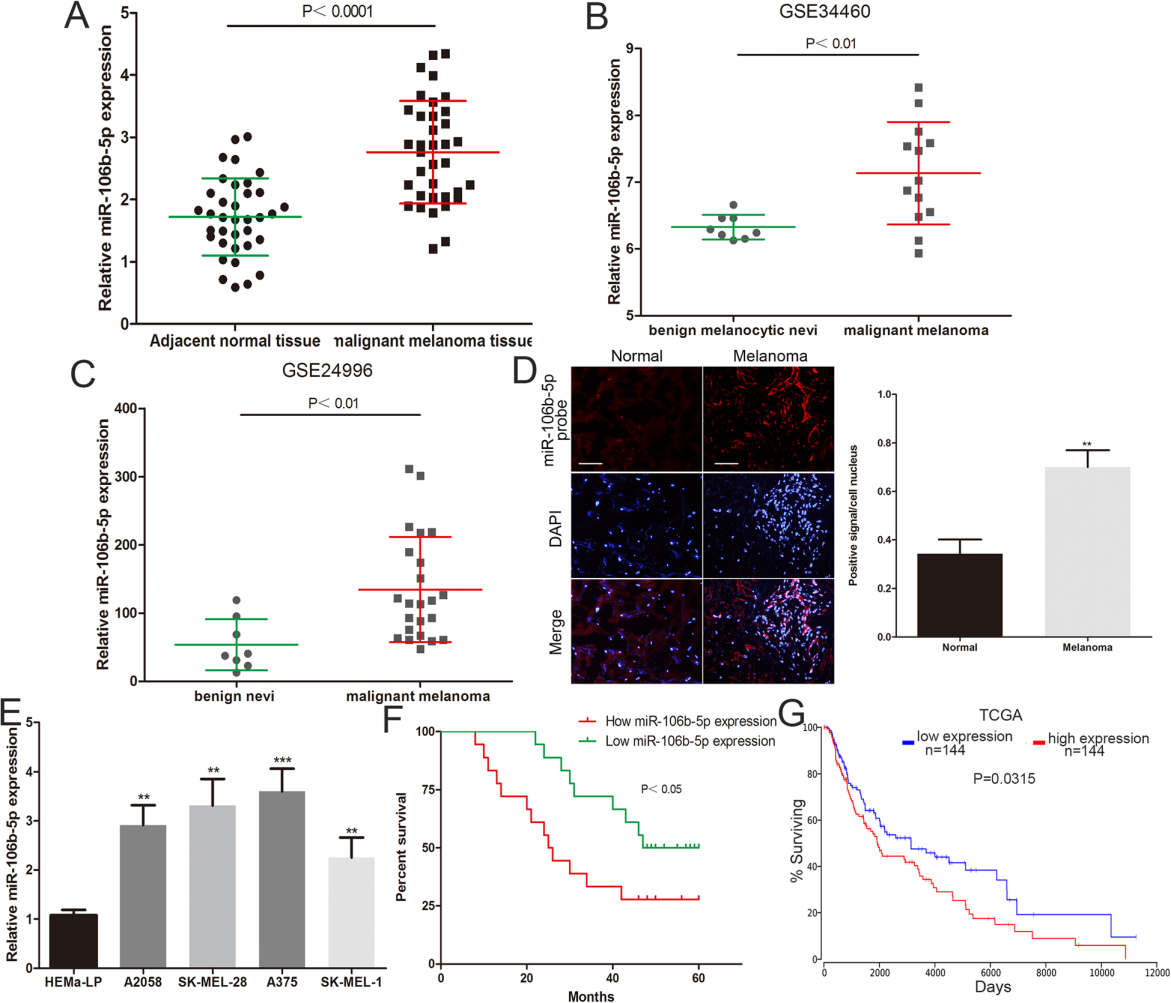
miR-106b-5p expression is upregulated in melanoma and an independent risk factor for the survival of patients with melanoma. **a** The expression of miR-106b-5p was detected in 36 malignant melanoma tissues and adjacent normal tissues. **b** The miR-106b-5p levels was analyzed by using GEO#GSE34460 dataset. **c** The miR-106b-5p levels was analyzed through GEO#GSE24996 dataset. **d** FISH analysis of miR-106b-5p in malignant melanoma tissues and adjacent normal tissues. Scale bar, 100 μm. **e** The miR-106b-5p expression profile in human melanoma cell lines (A375, A2058, SK-MEL-28, SK-MEL-1) and human epidermal melanocytes (HEMa-LP). **f** The overall survival curves of melanoma patients with high miR-106b-5p levels and low miR-106b-5p levels. **g** The prognostic data of melanoma in TCGA by using OncoLnc (http://www.oncolnc.org). Data were expressed as the mean ± SD, *P < 0.05, **P < 0.01, ***P < 0.001

First, we analysed miR-106b-5p expression levels in 36 malignant melanoma tissues and adjacent normal tissues by using qRT-PCR. We identified that miR-106b-5p expression was increased in melanoma tissue compared to matched normal tissues (Fig. 1a). The same results were confirmed by analysing previously published datasets (GEO#GSE34460 and GEO#GSE24996) (Fig. 1b and c). Moreover, FISH analysis showed a higher miR-106b-5p expression level in 5 selected melanoma tissues than in adjacent normal tissues (Fig. 1d). Malignant melanoma cells (A375, A2058, SK-MEL-1 and SK-MEL-28 cells) expressed higher miR-106b-5p levels than human epidermal melanocytes (HEMa-LP cells) (Fig. 14). Kaplan-Meier analysis showed that patients with melanoma with high miR-106b-5p expression (expression ratio ≥ median ratio) had poorer survival (Fig. 1f). We also analyzed the prognostic data of melanoma in TCGA by using OncoLnc (http://www.oncolnc.org), and found that the survival of melanoma patients with high miR-106b-5p expression (*n* = 144, miR-106b-5p expression accounted for the top 33% of 438 samples) was poorer than the low miR-106b-5p expression group (*n* = 144, miR-106b-5p expression accounted for the last 33% of 438 samples) (Fig. 1g). Furthermore, a high miR-106b-5p expression level was shown to be related to the clinical stage of melanoma but not to the age, sex, family history and occurrence of ulcers (Table 1). Univariate and multivariate Cox regression analyses confirmed that high miR-106b-5p expression is an independent risk factor for the overall survival of patients with melanoma (Table 2). These results suggested that miR-106b-5p may be involved in the malignant progression of melanoma.

**Table 1:** Correlation between miR-106b-5p expression and clinical pathological characteristic (*n* = 36)

Clinical characteristics	Number	High miR-106b-5p expression	Low miR-106b-5p expression	***P***-value
**Age**				0.729
<50	13	7	6	
≥ 50	23	11	12	
**Gender**				0.310
Male	21	12	9	
Female	15	6	9	
**Family history**				0.371
Yes	6	2	4	
No	30	16	14	
**TMN stage**				<0.01
I-II	14	3	11	
III	22	15	7	
**Ulcer**				0.502
Yes	20	9	11	
No	16	9	7

**Table 2:** Univariate and Multivariate Cox regression analysis of miR-106b-5p associated with overall survival rate in melanoma patients

**Univariate analysis**	**Hazard ratio**	**95% CI**	***P*-value**
**miR-106b-5p expression (high vs. low)**	2.517	1.057–5.992	0.037
**Multivariate analysis**	**Hazard ratio**	**95% CI**	***P*-value**
**miR-106b-5p expression (high vs. low)**	2.403	1.020–5.658	0.045

### miR-106b-5p is enriched in melanoma cell-secreted exosomes and transferred to melanocytes

**Fig. 2:**
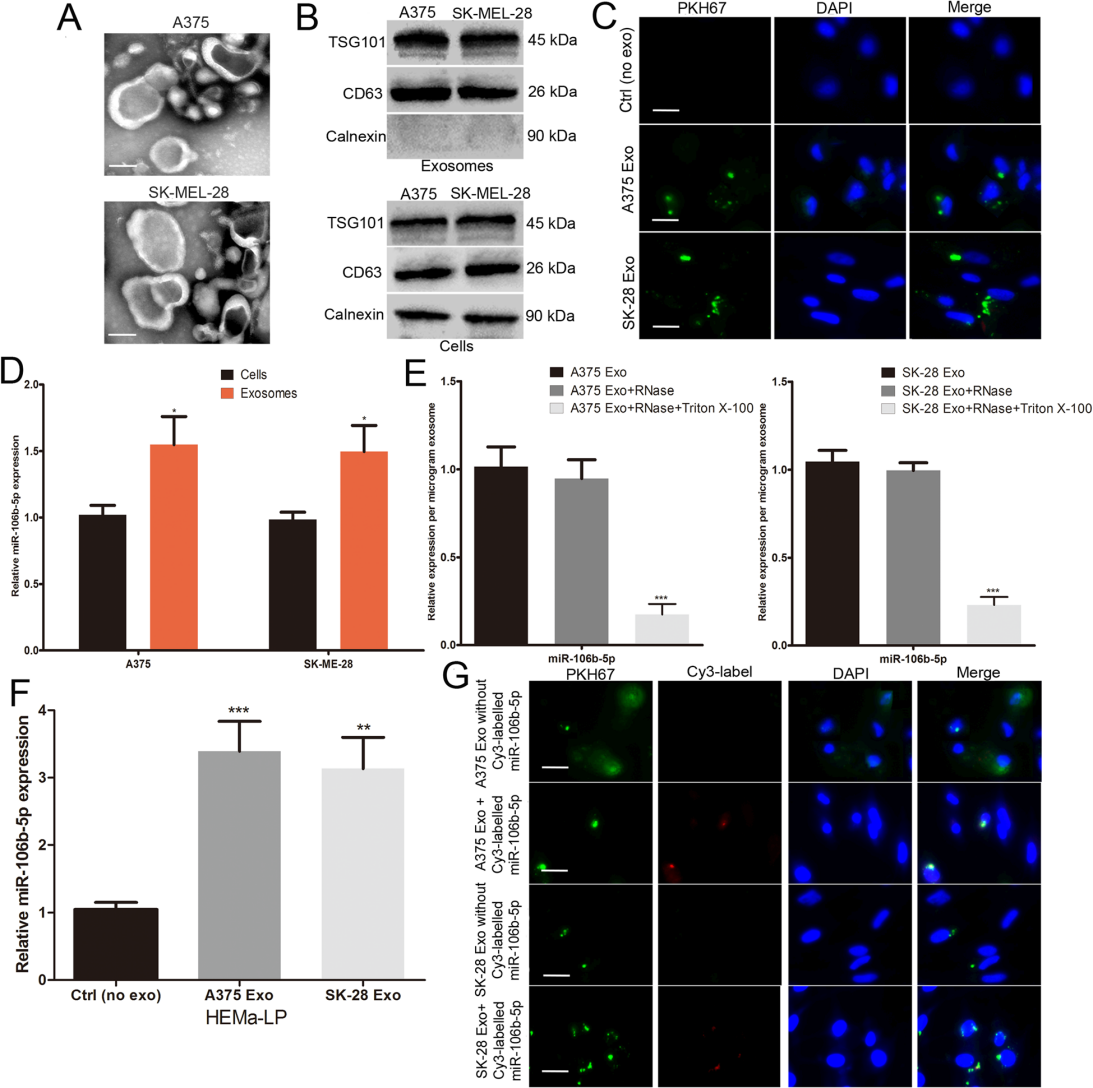
miR-106b-5p is enriched in melanoma cell-secreted exosomes and transferred to melanocytes. **a** Exosomes purified from culture supernatant of A375 and SK-MEL-28 cells were detected by TEM. Scale bar, 50 nm. **b** Western blots identified the exosomes marker proteins CD63 and TSG101, and Calnexin was used as an internal reference. **c** Exosomes purified from culture supernatant of A375 and SK-MEL-28 cells were labeled by PKH67. HEMa-LP cells was co-cultured with these exosomes and observed under confocal microscope, non-exosomes group was used as the negative control. Scale bar, 20 μm. **d** Basic miR-106b-5p levels in melanoma cells and paired exosomes were detected by qRT-PCR. **e** miR-106b-5p expression in exosomes, untreated or treated with RNase A and/or Triton X-100. **f** miR-106b-5p levels in HEMa-LP cells pre-treated with non-exosomes or indicated exosomes for 24 h were detected by qRT-PCR. **g** Exosomes with Cy3-labeled miR-106b-5p were added to HEMa-LP cells, fluorescence signals were detected under confocal microscope. miR-106b-5p without Cy3-label was used as the negative control. Scale bar, 20 μm. Data were expressed as the mean ± SD, *P < 0.05, **P < 0.01, ***P < 0.001

Previous studies have suggested that abundant miRNAs encapsulated in exosomes and play an important role in cell-to-cell communication [25, 26]. In addition, serum exosomal miR-106b-5p expression levels were increased in patients with melanoma [16]. Therefore, we investigated whether melanoma cell-derived exosomal miR-106b-5p is involved in the malignant progression of melanoma. Exosomes were initially isolated from two malignant melanoma cell lines (A375 and SK-MEL-28 cells). As shown in Fig. 2a, transmission electron microscopy (TEM) showed that exosomes had a typical cup-shaped morphology and were approximately 30–100 nm in diameter. Western blotting showed the presence of positive exosomal markers (CD63 and TSG101) in both the exosomal and cell fractions (Fig. 2b). Calnexin was used as a negative control, which was confirmed to be absent in the exosomes but present in the cells [27] (Fig. 2b). PKH67 (green) and 4′,6-diamidino-2-phenylindole (DAPI) (blue) were used to label the exosomes and cell nuclei, respectively. HEMa-LP human epidermal melanocytes were co-cultured with purified exosomes. PKH67-labelled exosomes were dispersed in the cytoplasm of HEMa-LP cells (Fig. 2c).

Furthermore, we found that miR-106b-5p was enriched in melanoma cell-secreted exosomes relative to their cellular content (Fig. 2d). The level of exosomal miR-106b-5p expression did not change upon RNaseA treatment but decreased after treatment with RNaseA and Triton X-100, indicating that extracellular miR-106b-5p was encapsulated (Fig. 2e). To confirm that melanoma cell-secreted exosomal miR-106b-5p can be transferred to HEMa-LP cells, we analysed the miR-106b-5p expression levels in HEMa-LP cells. The cellular expression levels of miR-106b-5p were increased in HEMa-LP cells following the treatment with melanoma cell-secreted exosomes (Fig. 2f). We transfected A375 and SK-MEL-28 cells with Cy3-labelled miR-106b-5p (red), and exosomes from A375 and SK-MEL-28 cells were then isolated and added to HEMa-LP cell culture supernatant. Red fluorescent signals were observed in the Cy3-labelled group but not in the negative control group (Fig. 2g). Overall, these results demonstrated that exosomal miR-106b-5p can be transferred from melanoma cells to HEMa-LP cells, leading to a significant increase in miR-106b-5p expression levels.

### Melanoma cell-secreted exosomal miR-106b-5p promotes the EMT of melanocytes

**Fig. 3:**
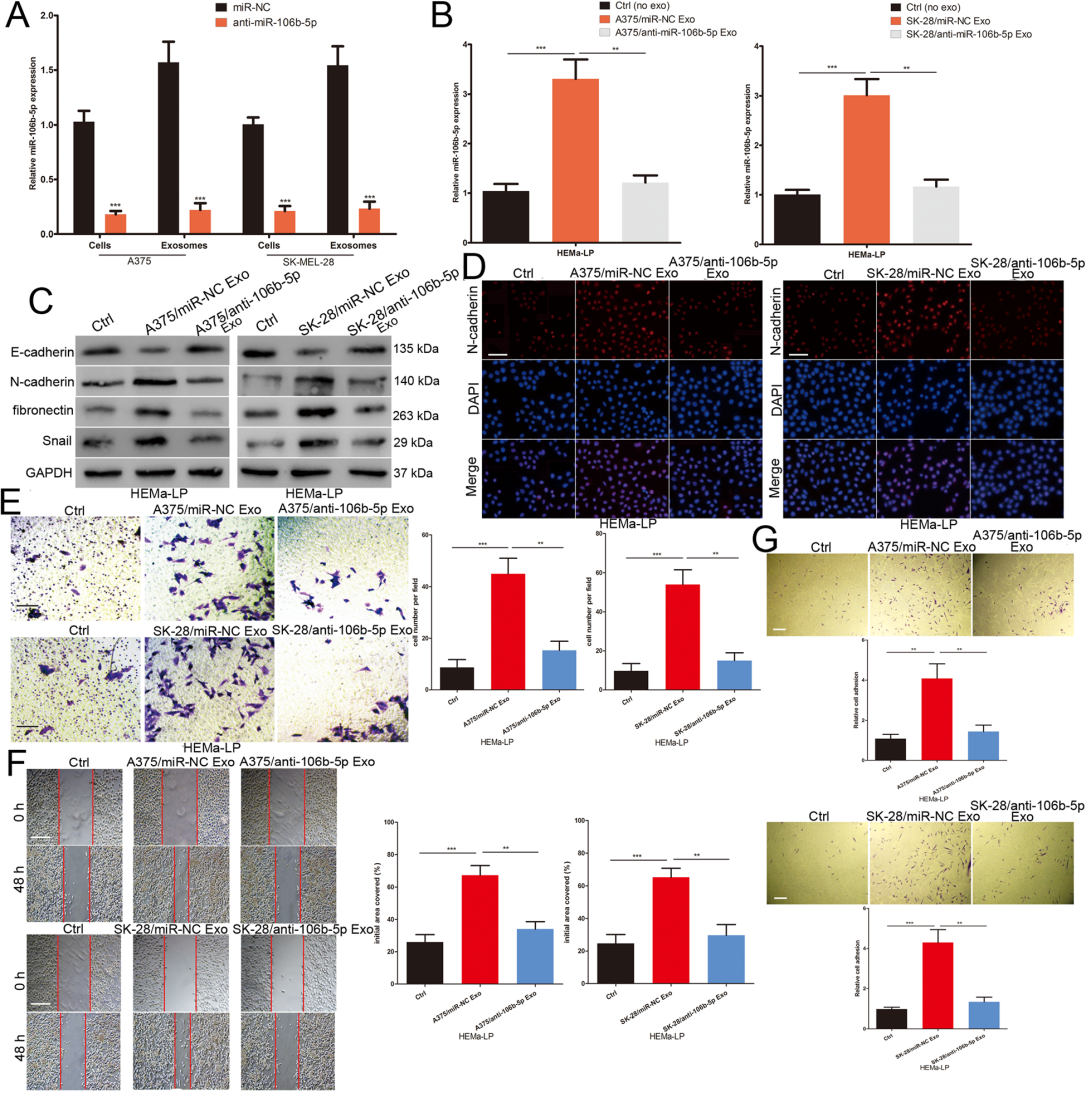
Melanoma cell-secreted exosomal miR-106b-5p promotes the EMT of melanocytes. **a** Cellular and exosomal miR-106b-5p levels in melanoma cells stably transfected with miR-106b-5p inhibitor or NC lentivectors were detected by qRT-PCR. **b** miR-106b-5p levels in HEMa-LP cells pre-treated with non-exosomes or melanoma exosomes with different treatment factors. **c** Western blots identified E-cadherin, N-cadherin, fibronectin and Snail protein expression changes in HEMa-LP cells treated with non-exosomes or indicated exosomes, GAPDH was used as a control. **d** N-cadherin were identified by immunofluorescence assays in HEMa-LP cells treated with non-exosomes or indicated exosomes. Scale bar, 100 μm. **e** The invasive capacity of HEMa-LP cells treated with non-exosomes or indicated exosomes was assessed by the transwell assay. Scale bar, 100 μm. **f** Migration capacity of HEMa-LP cells in different treatment groups was monitored by the scratch wound assay. Scale bar, 200 μm. **g** The ability of HEMa-LP cells adhesion to fibronectin was detected by the adhesion assay. Scale bar, 100 μm. Data were expressed as the mean ± SD, *P < 0.05, **P < 0.01, ***P < 0.001

To further investigate the role of exosomal miR-106b-5p in melanocytes, a lentiviral vector silencing miR-106b-5p or NC was transfected into A375 and SK-MEL-28 cells. Cellular and exosomal miR-106b-5p expression levels were significantly lower in anti-miR-106b-5p-transfected A375 and SK-MEL-28 cells compared with miR-NC-transfected A375 and SK-MEL-28 cells (Fig. 3a). HEMa-LP cells were incubated with exosomes secreted by miR-NC- and anti-miR-106b-5p-transfected melanoma cells. The expression levels of miR-106b-5p in HEMa-LP cells were increased in the miR-NC group, but there was no significant change in the anti-miR-106b-5p group (Fig. 3b). The expression level of the epithelial protein (E-cadherin) was decreased, whereas the mesenchymal proteins (N-cadherin and fibronectin) and EMT-TF (Snail) were increased in HEMa-LP cells incubated with exosomes secreted by miR-NC-transfected melanoma cells (Fig. 3c). Immunofluorescence staining of N-cadherin also showed stronger expression in HEMa-LP cells after miR-NC-transfected melanoma cell exosomes were added (Fig. 3d). The Transwell and scratch wound assays showed that exosomes secreted by miR-NC-transfected melanoma cells promoted the invasive and migratory ability of HEMa-LP cells (Fig. 3e and f). We also demonstrated that miR-NC-transfected melanoma cell-derived exosomes enhanced HEMa-LP cell adhesion to fibronectin (Fig. 3g). However, exosomes secreted by anti-miR-106b-5p-transfected A375 and SK-MEL-28 cells had no significant effect on the EMT, migration, invasion and adhesion of HEMa-LP cells (Fig. 3c-g). Overall, these data showed that melanoma cell-derived exosomal miR-106b-5p enhances melanocyte EMT, migration, invasion and adhesion.

### EphA4 is a direct target of miR-106b-5p

**Fig. 4:**
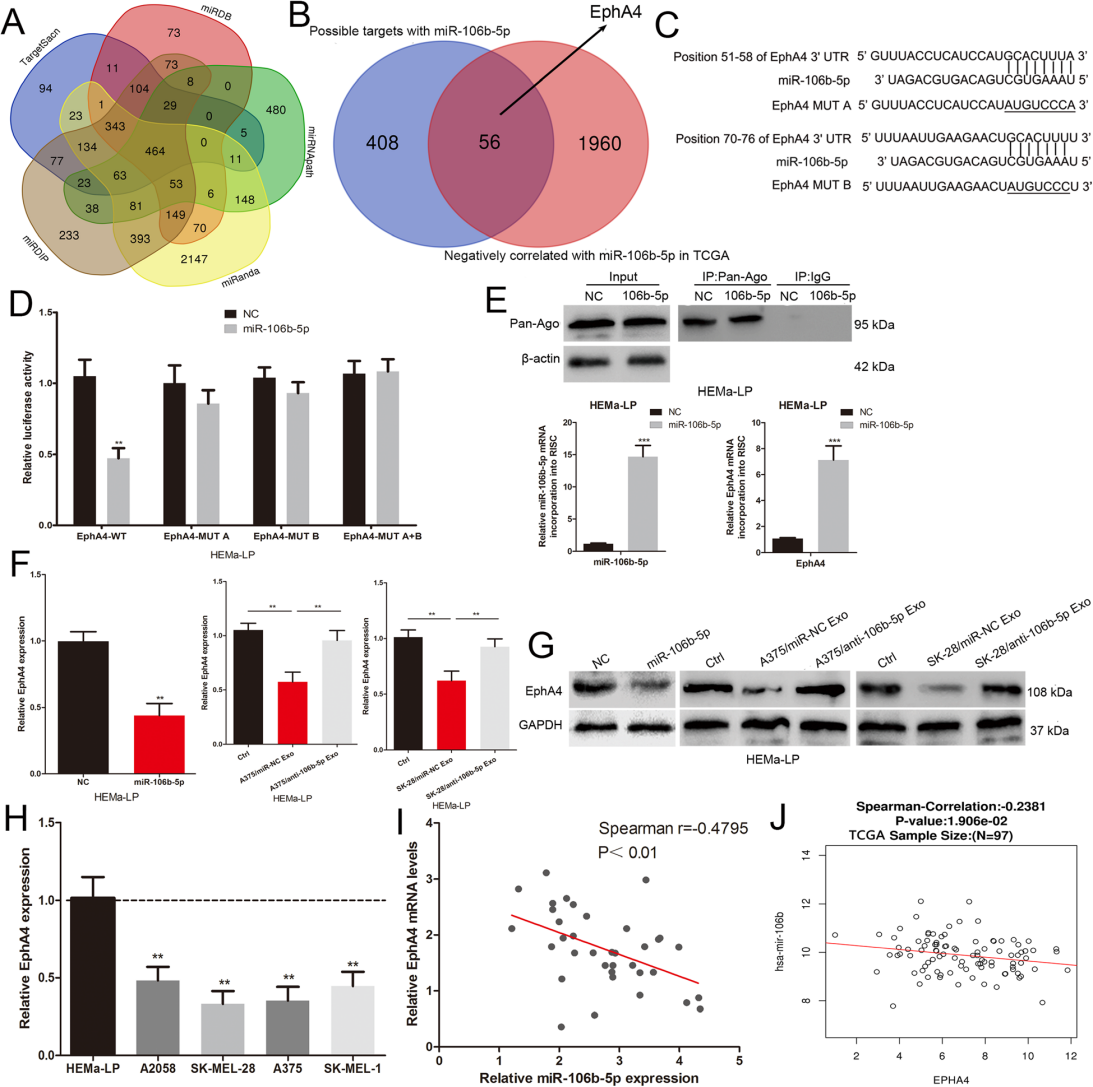
EphA4 is a direct target of miR-106b-5p. **a** Bioinformatics software (miRNApath, TargetScan, miRDIP, miRanda and miRDB) predict the potential target genes of miR-106b-5p. **b** Overlapping candidates with genes that are negatively correlated with miR-106b-5p in the TCGA database. **c** The binding sites of miR-106b-5p on the 3′-UTR of EphA4, and target sequences of EphA4–3′ UTRs were mutated. **d** Luciferase assay of cells transfected with EphA4–3′UTR-WT or EphA4–3′UTR-MUT reporter together with miR-106b-5p. **e** Immunoprecipitation of the Ago2/RISC using the Pan-Ago2 antibody in HEMa-LP cells overexpressing miR-106b-5p. IgG was used as a negative control and β-actin was used as an internal control. qRT-qPCR analysis of miR-106b-5p and EphA4 incorporated into RISC in HEMa-LP cells overexpressing miR-106b-5p compared to the levels in the control. U6 and GAPDH was used as an internal control. **f** RNA levels of EphA4 were detected by qRT-PCR in HEMa-LP cells treated with indicated exosomes or miR-106b-5p mimic. **g** Western blot analysis identified EphA4 protein expression changes in HEMa-LP cells treated with indicated exosomes or miR-106b-5p mimic. GAPDH was used as a control. **h** The EphA4 expression profile in human melanoma cell lines (A375, A2058, SK-MEL-28, SK-MEL-1) and human epidermal melanocytes (HEMa-LP). **i** The correlation of EphA4 mRNA and miR-106b-5p expression in 36 melanoma tissues was negative. **j** TCGA dataset revealed a significant negative correlation between EphA4 mRNA and miR-106b-5p in melanoma. Data were expressed as the mean ± SD, *P < 0.05, **P < 0.01, ***P < 0.001

To investigate how miR-106b-5p exerts its function in melanocytes, we used bioinformatics software to predict potential target genes. As predicted by miRNApath, TargetScan, miRDIP, miRanda and miRDB, 464 genes were found to have possible targets with miR-106b-5p (Fig. 4a). We narrowed the miR-106b-5p target genes to 56 genes by overlapping these candidates with genes that are negatively correlated with miR-106b-5p in the TCGA database (Fig. 4b). Among these candidates, EphA4 was shown to function as a negative regulator of the EMT and metastasis of melanoma cells and was chosen for further study [21]. We also verified the negative role of EphA4 in migration, invasion and adhesion of HEMa-LP cells (Supplementary Fig. 1A-C). We constructed EphA4 luciferase reporter vectors containing wild-type and mutant miR-106b-5p binding sites (Fig. 4c). The miR-106b-5p mimic led to a marked decrease in the luciferase activity of the wild-type EphA4 3’UTR plasmid relative to the mutant vector in HEMa-LP cells (Fig. 4d). We used RNA-ChIP analysis to analyse EphA4 mRNA abundance in the Ago2/RNA-induced silencing complex (RISC) after miR-106b-5p overexpression (Fig. 4e). Enrichment in the expression level of miR-106b-5p and EphA4 that was incorporated into RISC were observed in HEMa-LP cells with miR-106b-5p overexpression using RT-qPCR (Fig. 4e). The miR-106b-5p mimic and exosomes derived from the miR-NC-transfected A375 and SK-MEL-28 cells but not from the anti-miR-106b-5p-transfected A375 and SK-MEL-28 cells resulted in a decrease in EphA4 expression in HEMa-LP cells at the RNA and protein levels (Fig. 4f and g). Furthermore, melanoma cells (A375, A2058, SK-MEL-28 and SK-MEL-28) expressed lower EphA4 levels compared to human epidermal melanocytes (HEMa-LP) (Fig. 4h). Spearman’s correlation between the miR-106b-5p and EphA4 mRNA levels was negative in 36 melanoma tissues (Fig. 4i). TCGA datasets also revealed a statistically significant inverse correlation between miR-106b-5p and EphA4 transcript expression (Fig. 4j). All results indicated that EphA4 is the direct target of miR-106b-5p.

### Exosomal miR-106b-5p promotes the EMT of melanocytes by targeting EphA4 to activate the ERK pathway

**Fig. 5:**
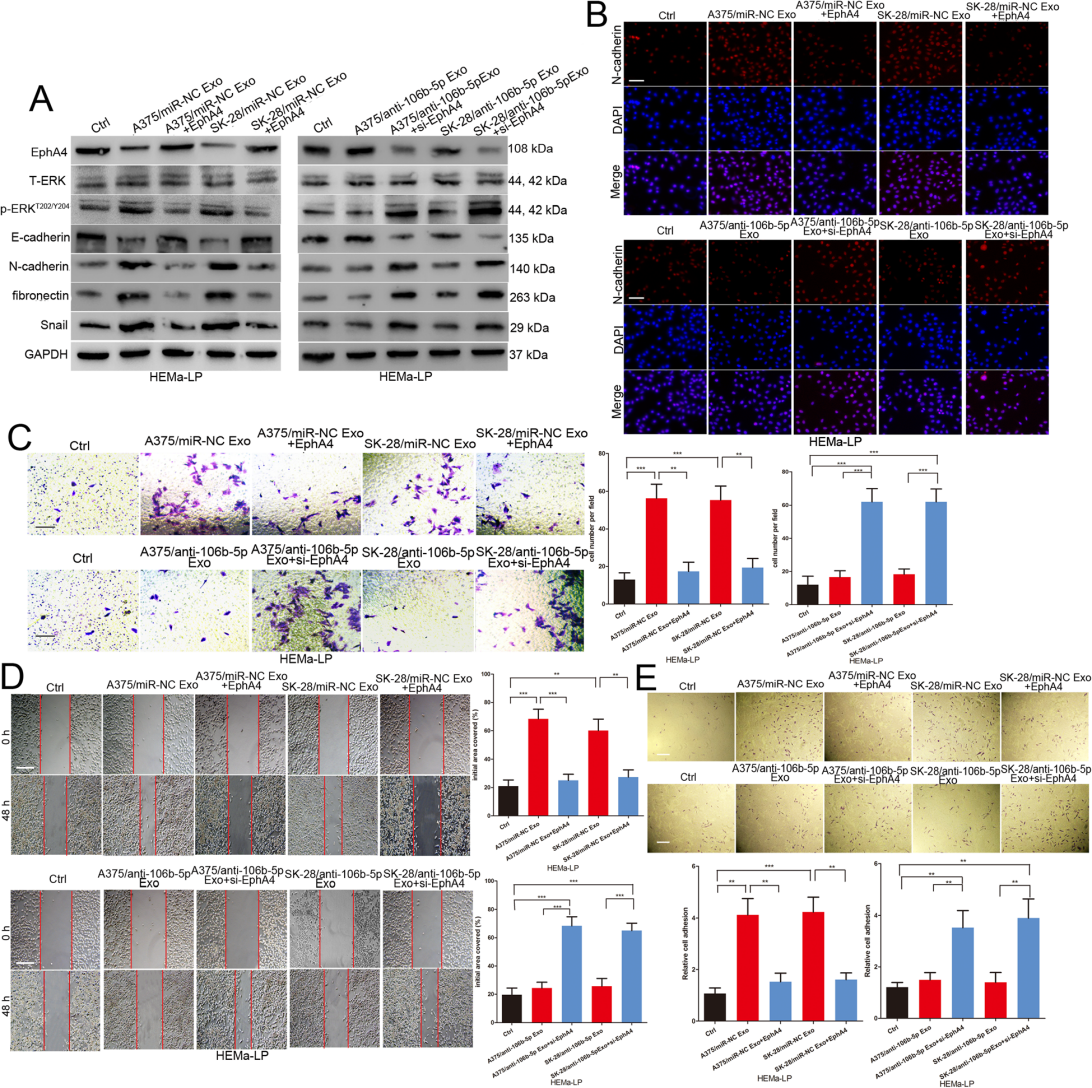
Exosomal miR-106b-5p promotes the EMT of melanocytes by targeting EphA4 to activate the ERK pathway. **a** Western blot analysis of EphA4, E-cadherin, N-cadherin, fibronectin and Snail, total ERK and p-ERK^T202/Y204^ in HEMa-LP treated with indicated exosomes in the presence of EphA4 plasmid or EphA4 siRNA, GAPDH was used as a control. **b** N-cadherin were identified by immunofluorescence assays in HEMa-LP cells treated with indicated exosomes in the presence of EphA4 plasmid or EphA4 siRNA. Scale bar, 100 μm. **c** The invasive capacity of HEMa-LP cells treated with indicated exosomes in the presence of EphA4 plasmid or EphA4 siRNA was assessed by the transwell assay. Scale bar, 100 μm. **d** Migration ability of HEMa-LP cells in different treatment groups was detected by the scratch wound assay. Scale bar, 200 μm. **e** The ability of HEMa-LP cells in different treatment groups adhesion to fibronectin was detected by the adhesion assay. Scale bar, 100 μm. Data were expressed as the mean ± SD, *P < 0.05, **P < 0.01, ***P < 0.001

EphA4 could interfere with the activation of ERK, which is a major pathway that mediates melanoma cell migration and invasion [21, 28]. We found that exosomes with high miR-106b-5p expression levels led to increased phosphorylated ERK expression in HEMa-LP cells (Fig. 5a). Furthermore, we explored whether exosomal miR-106b-5p induces melanocyte EMT by mediating EphA4 expression. We re-expressed EphA4 in HEMa-LP cells incubated with miR-NC-transfected A375 and SK-MEL-28 cell-derived exosomes and silenced EphA4 expression in HEMa-LP cells incubated with anti-miR-106b-5p-transfected A375 and SK-MEL-28 cell-derived exosomes (Fig. 5a). The EphA4 plasmid abrogated miR-NC-derived A375 and SK-MEL-28 cell-derived exosome-mediated promotion of the EMT, migration, invasion, adhesion and ERK pathway activation of HEMa-LP cells (Fig. 5a-e). EphA4 siRNA could provoke the abilities of anti-miR-106b-5p-transfected A375 and SK-MEL-28 cell-derived exosomes to promote the EMT, migration, invasion, adhesion and ERK pathway activation of HEMa-LP cells (Fig. 5a-e). Collectively, these results indicated that exosomal miR-106b-5p induces the EMT of melanocytes by regulating the EphA4/ERK pathway.

### Exosomal miR-106b-5p promotes melanoma metastasis in vivo

**Fig. 6:**
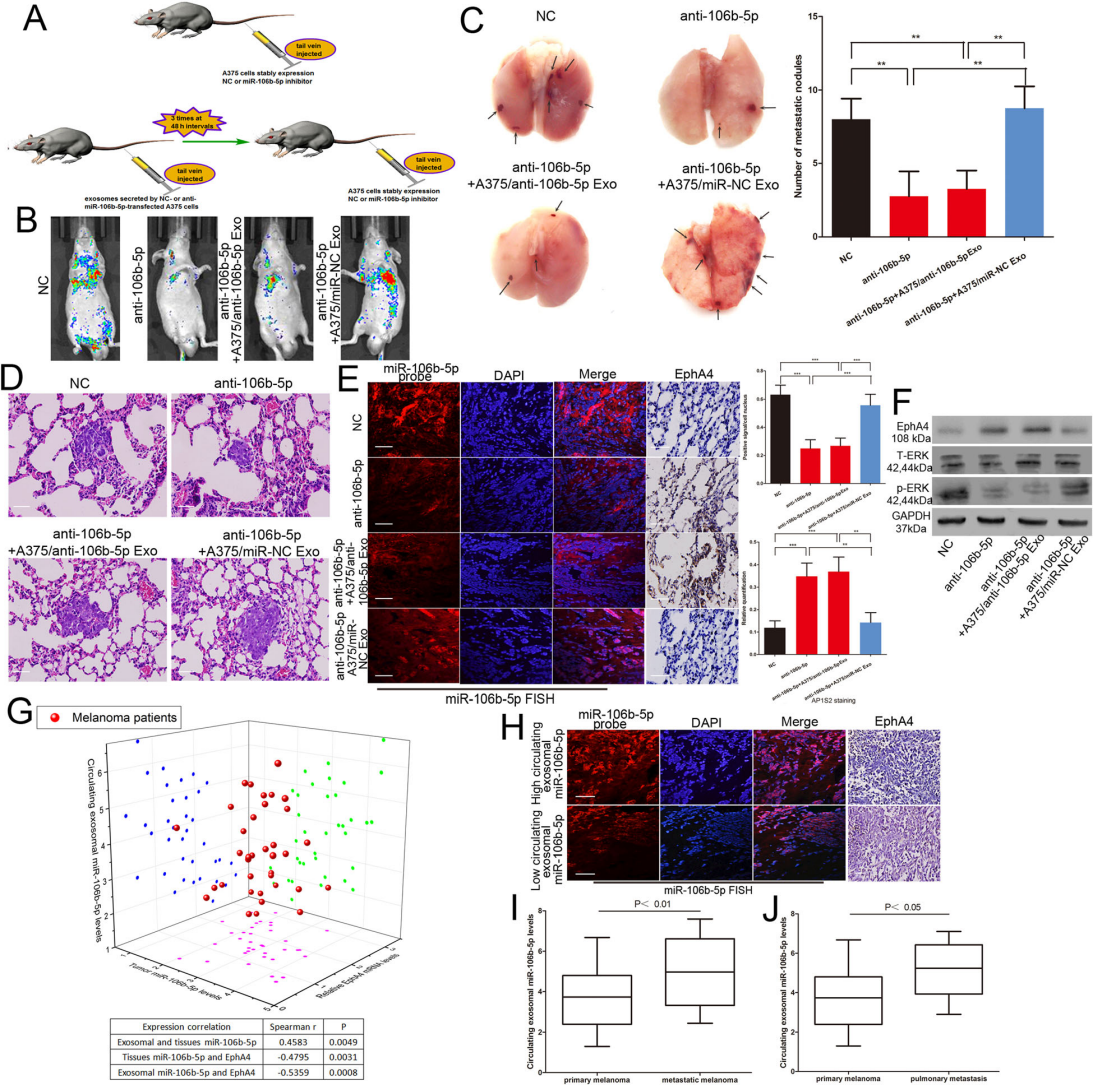
Exosomal miR-106b-5p promotes melanoma metastasis in vivo. **a** The schema of the animal experiment. **b** Representative bioluminescence images of mice after tail vein injection of stably expressing miR-106b-5p inhibitor A375 cells with or without intravenous injection of exosomes secreted by A375 cells. **c** The excision lung tissues in nude mice and the number of metastatic lung nodules. **d** Metastatic lung nodules were confirmed by H&E staining. Scale bar, 25 μm. **e** The expression of miR-106b-5p and EphA4 were detected by FISH and immunohistochemistry of sections from the metastatic lung nodules. Scale bar, 25 μm. **f** Western blot analysis of EphA4, total ERK and p-ERK^T202/Y204^ in metastatic lung nodules, GAPDH was used as a control. **g** Three dimensional scatter plot of circulating exosomal miR-106b-5p expression, tumour miR-106b-5p levels and EphA4 expression in 36 melanoma patients. **h** The expression of miR-106b-5p and EphA4 were detected by FISH and immunohistochemistry of sections from the malignant melanoma tissues. Scale bar, 25 μm. **i** The expression of circulating exosomal miR-106b-5p in metastatic and primary melanoma patients **j** the circulating exosomal miR-106b-5p level in 7 pulmonary metastasis patients and primary melanoma patients. Data were expressed as the mean ± SD, *P < 0.05, **P < 0.01, ***P < 0.001

We investigated the effect of miR-106b-5p on the invasive ability of melanoma cells in vivo. A375 cells transfected with the NC inhibitor lentivirus or miR-106b-5p inhibitor lentivirus were injected via the tail vein into nude mice (Fig. 6a). miR-106b-5p inhibitor-transfected A375 cells showed less lung colonization than the control group at 2 weeks post-injection (Fig. 6b). Knockdown of miR-106b-5p decreased the number of metastatic lung nodules (Fig. 6c). Nude mice were injected intravenously with 20 μg of exosomes secreted by miR-NC- or anti-miR-106b-5p-transfected A375 cells 3 times at 48 h intervals (Fig. 6a). miR-106b-5p inhibitor-transfected A375 cells were injected via the tail vein after exosome treatment (Fig. 6a). Mice preconditioned with intravenous miR-NC-transfected A375 cells-secreted exosome injections prior to the tail vein inoculation with the miR-106b-5p inhibitor-transfected A375 cells also showed a significant metastatic burden (Fig. 6b and c). Tumour tissues from metastatic lung nodules were confirmed by HE staining (Fig. 6d). We analysed relevant signalling molecule expression on sections of metastatic lung nodules. miR-106b-5p expression was increased and EphA4 expression was reduced in the NC inhibitor-transfected group and in the group pre-treated with exosomes secreted by miR-NC-transfected A375 cells (Fig. 6e). We also found that the overexpression of miR-106b-5p was accompanied by activation of ERK pathway (Fig. 6f).

It has been reported that serum exosomal miR-106b-5p is expressed at significantly higher levels in patients with melanoma [16]. We found that the circulating exosomal miR-106b-5p expression level was positively correlated with tumour miR-106b-5p expression levels but negatively correlated with EphA4 expression (Fig. 6g). FISH analysis and IHC staining also showed that high expression levels of circulating exosomal miR-106b-5p were accompanied by high expression levels of tumour miR-106b-5p and reduced EphA4 expression (Fig. 6h). We finally detected the circulating exosomal miR-106b-5p level in primary and 12 metastatic melanoma patients, 7 of whom had pulmonary metastasis. The clinical characteristic of primary and metastatic malignant melanoma was showed in Table 3. The expression of circulating exosomal miR-106b-5p was increased in metastatic melanoma patients compared to primary melanoma patients, the same is true for patients with pulmonary metastases (Fig. 6i and j). These observations indicated that melanoma cell-secreted exosomal miR-106b-5p facilitates melanoma metastasis in vivo.

**Table 3:** The clinical characteristic of primary and metastatic malignant melanoma (*n* = 48)

Clinical characteristics	Number	Primary malignant melanoma (*n* = 36)	Metastatic malignant melanoma (*n* = 12)	***P***-value
**Age**				0.724
<50	16	13	3	
≥ 50	32	23	9	
**Gender**				0.735
Male	28	21	7	
Female	20	15	5	
**Family history**				0.658
Yes	8	6	2	
No	40	30	10	

## Discussion

Melanoma has a high tendency for early metastasis. EMT has implications for tumour cell invasion by triggering the loss of cell-cell adhesion [29, 30]. Melanocytes express E-cadherin even though they are not epithelial cells [17]. The transformation of melanocytes to melanoma cells involves a series of genetic and environmental changes, and the loss of E-cadherin is the most important change [31]. Many studies have reported that EMT-like processes contribute to melanoma metastasis [17, 18]. Tumour cell-derived exosomes can carry oncogenic molecules, which can transfer to normal recipient cells and promote precancerous transformation [18, 32, 33]. Tumour-derived exosomes act as tumour messengers for intercellular crosstalk in the microenvironment and promote the growth and progression of tumours. For instance, breast cancer-derived exosomes induce a myofibroblast phenotype [34]. Exosomes derived from gastric cancer promotes peritoneal metastasis via mesothelial-to-mesenchymal transition [35]. Exosomes also play an important role in the malignant progression of melanoma [36, 37], but there are few studies reporting melanoma cell exosomes inducing EMT in the tumour microenvironment. In this study, we isolated exosomes from two malignant melanoma cell lines and analysed them by TEM and Western blot. Melanocytes acquire EMT characteristics after communicating with melanoma cell-derived exosomes.

Some specific miRNAs could be transferred into recipient cells through cancer cell-secreted exosomes and play a role by reducing the expression of target genes [13]. Liver cancer cell-derived exosomes transfer miR-1247-3p to recipient cells and induce CAFs by targeting B4GALT3, eventually leading to lung dissemination [38]. Exosomal miR-221-3p secreted by cervical squamous cell carcinoma promotes lymphatic metastasis by targeting VASH1 [39]. Only a few exosomal miRNAs have been identified as playing a role in the development of melanoma [36, 40]. The expression levels of exosomal miRNAs in the plasma of patients with melanoma are very different, and serum exosomal miR-106b-5p expression is significantly higher in patients with melanoma [16]. However, the function and molecular mechanism of exosomal miR-106b-5p in melanoma remains unknown. Our findings demonstrated that miR-106b-5p expression is upregulated in melanoma tissues and that high miR-106b-5p expression levels are an independent risk factor for patients with melanoma. miR-106b-5p is enriched in melanoma cell-secreted exosomes and transferred to melanocytes. Melanoma cell-derived exosomal miR-106b-5p expression promotes the EMT, migration, invasion and adhesion of melanocytes.

EphA4, a member of the Eph receptor tyrosine kinase family, has been shown to play different roles in different human tumours. For instance, EphA4 promotes cell proliferation and migration of glioma cells through the FGFR1 signalling pathway [19]. In contrast, EphA4 reduces tumour cell migration and invasion in lung adenocarcinoma through EphA4-dependent ERK1/2 inactivation [20]. Moreover, EPHA4 has been reported to inhibit the EMT and metastasis of melanoma cells by interfering with ERK activation [21]. In this study, we demonstrated that EphA4 is targeted by miR-106b-5p. Exosomal miR-106b-5p promotes the EMT of melanocytes by repressing EphA4 to activate the ERK pathway. We also demonstrated that melanoma cell-secreted exosomal miR-106b-5p facilitates melanoma metastasis in vivo, but the results need further determination in a larger number of samples. In the future, we will collect more clinical samples of metastatic melanoma to further support our findings.

## Conclusion

In summary, the overexpression of miR-106b-5p may play a crucial role in the progression of melanoma. Furthermore, melanoma cell-derived exosomes transfer miR-106b-5p to melanocytes. miR-106b-5p targets the EphA4 to activate the ERK pathway and induces the EMT of melanocytes, established the microenvironment characteristics that facilitate tumor metastasis. Understanding the molecular biological basis of tumour-derived exosomes harbouring EMT regulators in melanoma is helpful to recognize new biomarkers and new potential therapeutics for melanoma.

## Supplementary Information

**Additional file 1: Supplementary Figure 1.** The role of EphA4 in melanocytes. (A) The invasive capacity of HEMa-LP cells was assessed by transwell assay. (B) Migration capacity of HEMa-LP cells in different treatment groups was monitored by scratch wound assay. (C) The ability of HEMa-LP cells adhesion to fibronectin was detected by adhesion assay. Data were expressed as the mean ± SD, *P < 0.05, **P < 0.01, ***P < 0.001.

## Data Availability

All the data and materials supporting the conclusions were included in the main paper.

## Abbreviations

qRT-PCR: Quantitative real-time polymerase chain reaction
TEM: Transmission electron microscopy
ChIP: Chromatin Immunoprecipitation
EMT: Epithelial-to-mesenchymal transition
EMT-TFs: Epithelial-to-mesenchymal transition inducing transcription factors
EphA4: Erythropoietin-producing hepatocellular carcinoma receptor A4
FBS: Foetal bovine serum
GEO: Gene Expression Omnibus
TCGA: The Cancer Genome Atlas
DMEM: Dulbecco’s modified Eagle’s medium
siRNAs: Small interference RNAs
RT: Reverse transcription
PBS: Phosphate-buffered saline
IF: Immunofluorescence
FISH: Fluorescence in situ hybridization
S.D.: Standard deviation
DAPI: 4′,6-diamidino-2-phenylindole
HE: Hematoxylin-eosin
