# Supplementary figures and images for: Exosomal miR-106b-5p derived from melanoma cell promotes primary melanocytes epithelial-mesenchymal transition through targeting EphA4
Source: J Exp Clin Cancer Res. 2021 Mar 19;40:107. doi: 10.1186/s13046-021-01906-w (PMC7980627; doi:10.1186/s13046-021-01906-w)

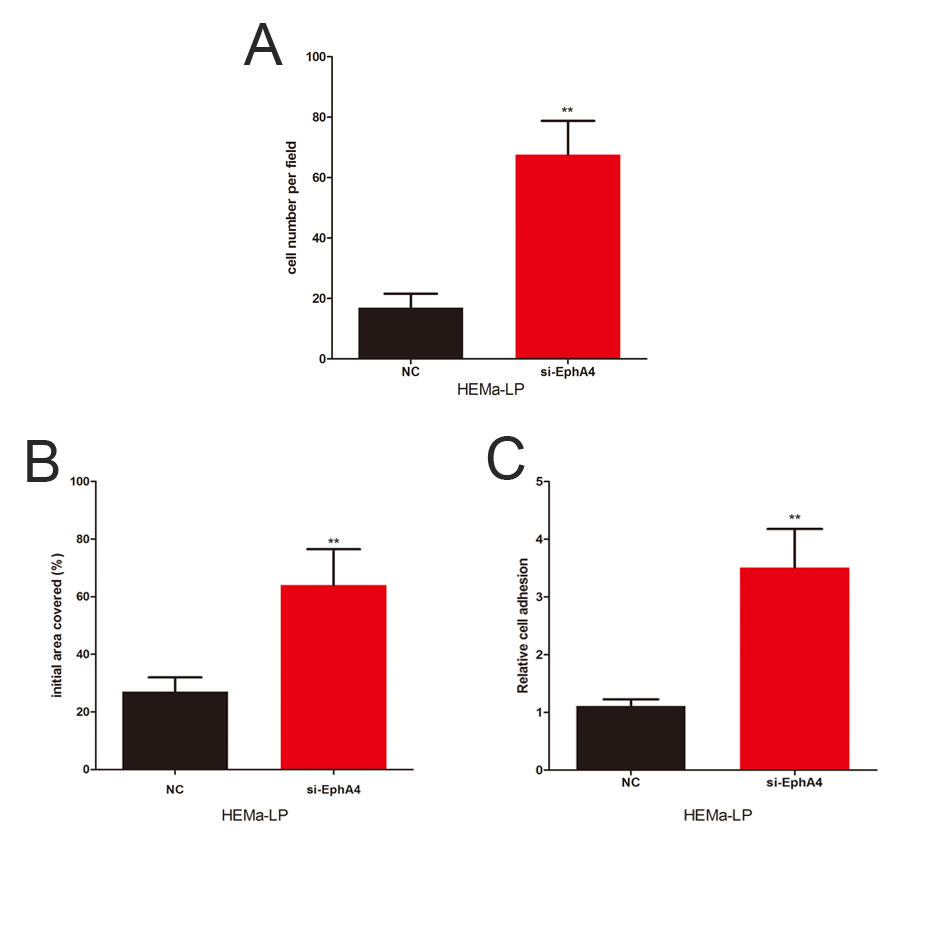

Supplement: Supplementary file 1 — Additional file 1: Supplementary Figure 1. The role of EphA4 in melanocytes. (A) The invasive capacity of HEMa-LP cells was assessed by transwell assay. (B) Migration capacity of HEMa-LP cells in different treatment groups was monitored by scratch wound assay. (C) The ability of HEMa-LP cells adhesion to fibronectin was detected by adhesion assay. Data were expressed as the mean ± SD, *P < 0.05, **P < 0.01, ***P < 0.001. [file 13046_2021_1906_MOESM1_ESM.tif]
